# The Impact of Vitreoretinal Surgery in Patients with Uveitis: Current Strategies and Emerging Perspectives

**DOI:** 10.3390/diagnostics16020198

**Published:** 2026-01-08

**Authors:** Dimitrios Kalogeropoulos, Sofia Androudi, Marta Latasiewicz, Youssef Helmy, Ambreen Kalhoro Tunio, Markus Groppe, Mandeep Bindra, Mohamed Elnaggar, Georgios Vartholomatos, Farid Afshar, Chris Kalogeropoulos

**Affiliations:** 1 Ophthalmology Department, Stoke Mandeville Hospital, Buckinghamshire Healthcare NHS Foundation Trust, Mandeville Rd, Aylesbury HP21 8AL, UK; m.latasiewicz@nhs.net (M.L.); y.helmy@nhs.net (Y.H.); a.tunio@nhs.net (A.K.T.); markus.groppe@nhs.net (M.G.); mandeep.bindra@nhs.net (M.B.); m.elnaggar2@nhs.net (M.E.); 2 Ophthalmology Department, Faculty of Medicine, School of Health Sciences, University of Thessaly, University Hospital of Larissa, 41110 Larissa, Greece; androudi@otenet.gr; 3 Kasr Al-Ainy, Faculty of Medicine, Cairo University, Cairo 4240310, Egypt; 4 Haematology Laboratory, Unit of Molecular Biology, University Hospital of Ioannina, 45500 Ioannina, Greece; gvarthol@gmail.com; 5 Southampton Eye Unit, University Hospital Southampton, Southampton SO16 6YD, UK; farid.afshar@uhs.nhs.uk; 6 Department of Ophthalmology, Faculty of Medicine, School of Health Sciences, University of Ioannina, 45500 Ioannina, Greece; kalogch@otenet.gr

**Keywords:** uveitis, intraocular inflammation, pars plana vitrectomy, diagnostic vitrectomy, therapeutic vitrectomy, vitreous analysis

## Abstract

Uveitis constitutes a heterogeneous group of intraocular inflammatory pathologies, including both infectious and non-infectious aetiologies, often leading to substantial morbidity and permanent loss of vision in up to 20% of the affected cases. Visual impairment is most prominent in intermediate, posterior, or panuveitis and is commonly associated with cystoid macular oedema, epiretinal membranes, macular holes, and retinal detachment. In the context of uveitis, these complications arise as a result of recurrent flare-ups or chronic inflammation, contributing to cumulative ocular damage. Pars plana vitrectomy (PPV) has an evolving role in the diagnostic and therapeutic approach to uveitis. Diagnostic PPV allows for the analysis of vitreous fluid and tissue using techniques such as PCR, flow cytometry, cytology, and cultures, providing further insights into intraocular immune responses. Therapeutic PPV can be employed for the management of structural complications associated with uveitis, in a wide spectrum of inflammatory clinical entities such as Adamantiades–Behçet disease, juvenile idiopathic arthritis, acute retinal necrosis, or ocular toxoplasmosis. Modern small-gauge and minimally invasive techniques improve visual outcomes, reduce intraocular inflammation, and may decrease reliance on systemic immunosuppression. Emerging technologies, including robot-assisted systems, are expected to enhance surgical precision and safety in the future. Despite these advances, PPV outcomes remain variable due to heterogeneity in indications, surgical techniques, and postoperative management. Prospective studies with standardized protocols, detailed subgroup analyses, and the integration of immunological profiling are needed to define which patients benefit most, optimize therapeutic strategies, and establish predictive biomarkers in uveitis management.

## 1. Introduction

Uveitis comprises an extremely diverse group of intraocular inflammatory pathologies associated with both infectious and non-infectious aetiologies [1,2]. Visual distortion is more frequent in patients with intermediate, posterior, or panuveitis and is usually associated with anatomical and structural disorders, such as cystoid macular oedema (CMO), epiretinal membranes (ERMs), macular holes (MH), and retinal detachment (RD) [3]. These complications and the associated visual impairment are typically a result of uveitis flare-ups and/or chronic inflammatory activity, leading to the so-called “cumulative damage,” a term coined by Quan Dong Nguyen in 2006 [4]. Recent studies have highlighted the pivotal role of therapeutic pars plana vitrectomy (PPV) in uveitis [5]. Apart from the surgical management of uveitis-related complications, PPV can contribute substantially to the diagnostic orientation of intraocular inflammation, especially in cases with diagnostic uncertainty (e.g., atypical infections or masquerade syndromes) [3]. Furthermore, it has been increasingly evident that removing the vitreous during diagnostic sampling or while managing complications in uveitis can contribute to improvements in various parameters of the intraocular inflammation [6,7,8,9]. These parameters include visual acuity, inflammatory score of aqueous and vitreous humour, cystoid macular oedema (CMO), and the need for long-term immunosuppression. The majority of published studies are retrospective, non-comparative interventional case series, whereas some prospective controlled studies and systematic literature reviews have also been conducted [6,7,8,10,11,12,13]. Despite these observations, PPV has not been routinely integrated into the management of intraocular inflammation. Its implementation may be discouraged by several factors, including concerns about operating on an inflamed eye, the high risk of complications, as well as the actual inability of the procedure itself to provide a definitive cure for the disease [9,14]. It has been suggested that the perceived benefits of therapeutic PPV may be only “pseudo-positive”, as improvements in visual acuity could simply arise from the removal of vitreous opacities or debris rather than any pragmatic reduction in the intraocular inflammatory activity. Others support that its efficacy may be a result of “clearing” of eyes with inactive uveitis that are no longer responding to traditional anti-inflammatory treatments. Therefore, the exact role of PPV in uveitis has not yet been fully established [9,14]. The specific indications, contraindications, and surgical protocols for its use remain vaguely defined in the current literature. Although systematic reviews on diagnostic and therapeutic PPV in uveitis have been published in the past, they are limited by variations in case indications, preoperative considerations, surgical techniques, and postoperative management strategies [6,8]. The aim of this narrative review is to approach the diagnostic and therapeutic applications of vitrectomy in uveitis. Particular emphasis is directed towards sophisticated diagnostic techniques, such as flow cytometry, as well as modern surgical techniques for the management of the complications caused by intraocular inflammation. Additionally, we provide tips and advice for the management of patients undergoing PPV in the context of uveitis.

## 2. Search Method

A narrative review of the literature was performed to assess the diagnostic and therapeutic implications of PPV in patients with uveitis. A comprehensive search of PubMed/MEDLINE, Embase, Scopus, and the Cochrane Library was conducted. The search covered publications from January 1972 to December 2025, reflecting both the earliest studies of PPV and recent advances in diagnostics and surgical techniques. The main keywords/search terms included combinations of “uveitis”, “pars plana vitrectomy”, “diagnostic vitrectomy”, therapeutic vitrectomy”, “vitreous biopsy”, “epiretinal membrane”, “cystoid macular oedema”, “macular hole”, “retinal detachment”, “vitreoretinal lymphoma”, “infectious uveitis”, “non-infectious uveitis”, “masquerade syndrome”, “polymerase chain reaction”, “flow cytometry”, “small-gauge vitrectomy”, “robotic surgery”, and “artificial intelligence”. Boolean operators (AND/OR) were used to refine searching, and the reference lists of relevant papers were manually checked to detect additional key studies.

Studies were included if they met one or more of the following criteria:

1. Evaluating the role of diagnostic PPV in uveitis and associated laboratory techniques (e.g., microbiological analysis, flow cytometry);
2. Addressing the therapeutic role of PPV for the management of uveitis-related complications (e.g., epiretinal membrane or retinal detachment);
3. Reporting outcomes of PPV in specific uveitic entities (e.g., ocular toxoplasmosis or acute retinal necrosis);
4. Reviewing surgical techniques, technological advances, or other aspects relevant to the management of uveitis.

Our analysis included original research articles, randomized and non-randomized clinical studies, cohort studies, case series, and high-quality narrative or systematic reviews published in English. Additionally, one article in German and one in French were also included due to their relevance to the topic. We selectively included case reports addressing uncommon indications, novel techniques, or rare complications. Articles unrelated to uveitis, non-vitrectomy surgical approaches, or lacking clinical relevance were excluded.

This narrative review was prepared with reference to the Scale for the Assessment of Narrative Review Articles (SANRA), and the manuscript was supplemented where appropriate to ensure methodological rigor.

## 3. Historical Perspective

In 1972, Machemer et al. [15] introduced the concept of pars plana vitrectomy (PPV) to the American Ophthalmological Society. Approximately a decade later, the first reports exploring the role of vitrectomy in uveitic patients appeared in the literature [16,17,18,19,20]. Over the years, technological advances and more sophisticated surgical techniques have refined PPV, expanding its applications in uveitis for both diagnostic and therapeutic purposes [21]. Traditionally, vitrectomy has often enhanced pharmacologic treatments by the surgical elimination of vitreous debris and opacities, and the management of structural abnormalities. Initial studies largely focused on infectious uveitis, particularly endophthalmitis, complicating the understanding and interpretation of early outcomes of studies for non-infectious uveitis [6,17,18,20,22]. In 1978, Diamond and Kaplan published one of the first reports on the application of vitreoretinal surgery in non-infectious uveitis; they described combined lensectomy-vitrectomy procedures in eyes with complex uveitis cataracts [23]. According to their results, there was reduced CMO, while other similar case series also reported favourable outcomes. It was hypothesized that removing the vitreous could reduce the load of inflammatory cells and immune complexes. However, it remains uncertain whether the vitreous humour contains antigens that can induce inflammatory activity or whether the vitritis represents a secondary phenomenon [20,23,24,25]. Algvere et al. [16] described 14 cases of patients with uveitis who underwent therapeutic vitrectomy. Their results demonstrated an improvement of vision in 10 of these cases, whereas there was a notable reduction in the inflammatory activity in the anterior chamber. A few years later, in 1984, Leuenberger [26] and Freyler [27] recorded 30 and 34 uveitic patients, respectively, with good functional outcome after PPV. These individuals had fewer and less severe recurrences, as well as reduced reliance on anti-inflammatory or immunosuppressive drugs. Furthermore, early reports underlined the diagnostic contribution of vitrectomy in collecting specimens [26,28,29,30]. Modern, less-invasive surgical techniques and advances in small-gauge instrumentation have aided in shorter procedures and faster recovery, thereby expanding their use in clinical practice. Nevertheless, even with these technological improvements, our understanding of PPV’s role in uveitis is still evolving [21].

## 4. The Diagnostic Applications of Vitreoretinal Surgery in Uveitis

**Figure 1:**
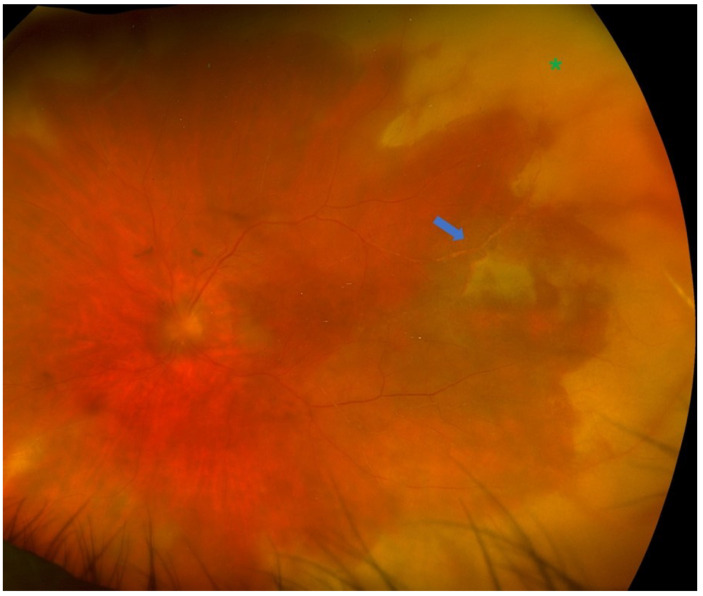
Optos colour widefield photography of the left eye in a healthy, immunocompetent 30-year-old male diagnosed with unilateral acute retinal necrosis secondary to Varicella Zoster Virus, confirmed by polymerase chain reaction (PCR) following a vitreous biopsy. The green asterisk marks the area of retinal necrosis, with associated haemorrhagic features noted in the superotemporal region, showing circumferential spread along the temporal retina. The blue arrow highlights an area of occlusive vasculopathy affecting the arterioles. Image courtesy of Mr. Farid Afshar, Consultant Ophthalmologist, University Hospital Southampton, UK.

**Figure 2:**
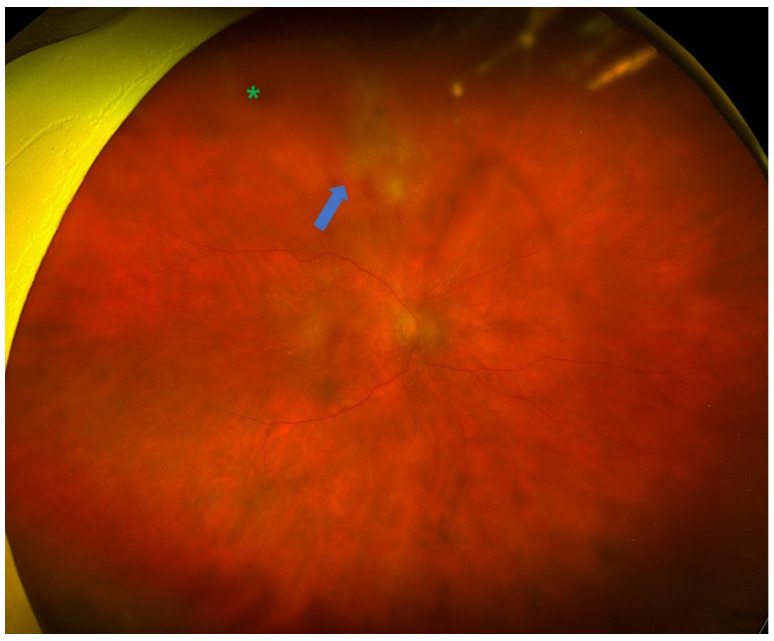
Optos widefield colour photography of the right eye in an otherwise healthy, immunocompetent 60-year-old female showing an area of cytomegalovirus (CMV) retinitis. The blue arrow indicates the focal area of retinitis, while the green asterisk denotes the associated overlying vitritis. Image courtesy of Mr. Farid Afshar, Consultant Ophthalmologist, University Hospital Southampton, UK.

**Figure 3:**
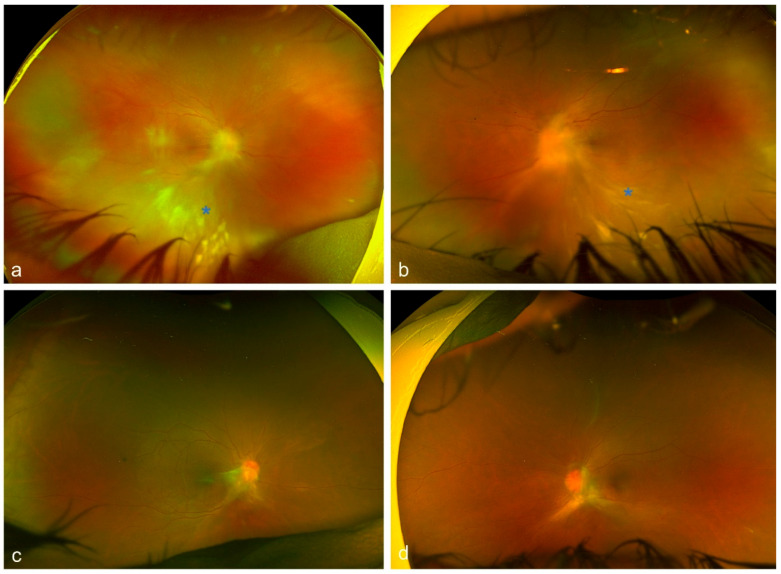
Optos widefield colour photography (**a**) right eye; (**b**) left eye demonstrating bilateral leukaemic infiltration (blue asterisks), possibly via the optic nerve, in a 27-year-old female with known acute myeloid leukaemia (AML). A diagnostic vitrectomy was performed in the right eye to exclude infectious causes in the context of immunosuppression. (**c**) (right eye) and (**d**) (left eye) show bilateral improvement following treatment with intravitreal methotrexate injections in conjunction with systemic therapy. Image courtesy of Mr. Farid Afshar, Consultant Ophthalmologist, University Hospital Southampton, UK.

Uveitis is classified as non-infectious or infectious, comprising a wide spectrum of aetiologies with complex pathogenetic mechanisms [1,2]. The exact aetiology is not always obvious, and many cases can cause diagnostic uncertainty due to the variety of clinical manifestations. Making an accurate and prompt diagnosis is essential for achieving positive functional and anatomical outcomes [3]. It has been shown that, in selected cases, the analysis of intraocular fluids and tissues [polymerase chain reaction (PCR), flow cytometry, cytology, and cultures] can provide substantial information and support diagnostic orientation [3]. Diagnostic vitrectomy is indicated in atypical cases of uveitis, when undetermined or unidentified pathogenic agents are involved, or in patients with poor response to treatment [31]. Indications for performing a diagnostic vitrectomy include infectious causes (e.g., vitritis, retinitis, choroiditis, or endophthalmitis) [Figure 1 and Figure 2], non-infectious causes (e.g., autoimmune uveitis), or malignant processes [e.g., primary vitreoretinal lymphoma (PVRL), metastasis, choroidal melanoma] [3,32,33] [Figure 3].

### 4.1. Vitreous Tap/Biopsy

Vitreous tap has been implemented as a diagnostic tool for cases with intraocular inflammation since the 1970s. This approach facilitates the collection of approximately 0.5–2 mL of vitreous humour, providing a greater sample volume compared to an aqueous tap. A vitreous tap is performed by inserting a 27- to 22-gauge needle attached to a tuberculin syringe through the pars plana into the vitreous cavity. When adequate fluid cannot be obtained, vitreous biopsy is recommended to avoid damage from aspirating formed vitreous [34]. The results of the Endophthalmitis Vitrectomy Study (EVS) indicated that, in eyes with postoperative endophthalmitis, vitreous samples yielded positive cultures more frequently than aqueous samples. Moreover, there was no substantial difference between vitreous tap and mechanised vitreous biopsy in terms of microbiologic yield, complication rate, visual outcomes, or short-term risk of RD [35]. Manku and McCluskey [36] conducted a retrospective study evaluating the vitreous biopsy results of 59 consecutive patients with posterior uveitis or panuveitis. They found that biopsy results critically influenced management in 12% of cases and confirmed or ruled out infection in 68%. In their cohort, postoperative complications were rare (only one case each of hypotony and retinal detachment). They concluded that vitreous biopsy is a safe and effective diagnostic method for confirming or excluding infectious and malignant aetiologies of intraocular inflammation.

### 4.2. Diagnostic Vitrectomy

Despite recent advances in modern vitrectomy techniques, obtaining a sufficient vitreous sample remains challenging. Considering that small sample volumes and poor preparation can significantly lessen diagnostic accuracy, the aim of a successful vitrectomy is to collect the largest possible amount of undiluted tissue while ensuring optimal sample handling. The diagnostic yield varies widely among different studies, as it depends on various factors such as patient selection, surgical methods, sample size, and the quality of sample analysis. Moreover, it is critical to underline that a negative result does not necessarily rule out infection or a malignancy [21].

**Figure 4:**
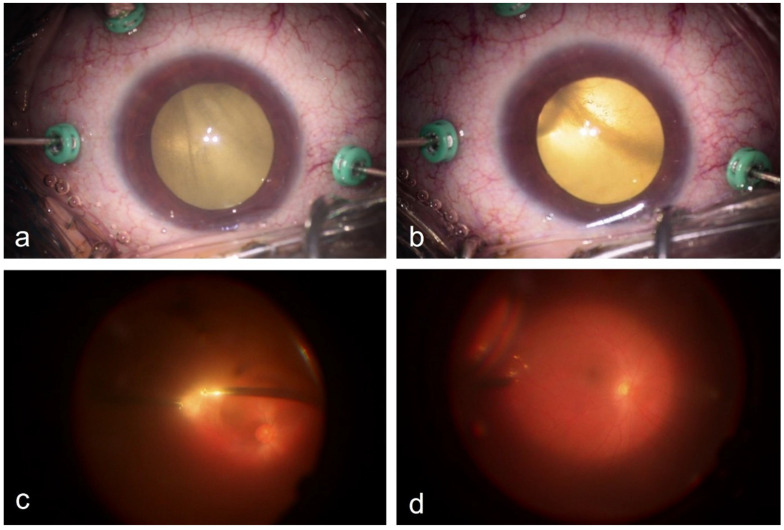
(**a**) During a diagnostic pars plana vitrectomy, a standard three-port pars plana approach is used with small-gauge instrumentation (23-, 25-, or 27-gauge). After trocar placement, the infusion line is connected but kept switched off to prevent dilution of the sample. (**b**) An undiluted vitreous sample (typically 0.5–1.0 mL) is first aspirated using the vitreous cutter. To prevent hypotony, the fluid–air exchange function on the vitrectomy machine is activated so that air fills the vitreous cavity and vitrectomy proceeds underneath. A syringe is attached to the cutter after disconnecting the vacuum tubing. (**c**) Following sample collection, balanced salt solution infusion is activated as is normal in vitrectomy, and a limited core vitrectomy is performed to obtain additional diluted vitreous if needed for supplementary testing. (**d**) Pars plana vitrectomy then proceeds as normal after reconnecting the vacuum tubing to the vitreous cutter. Some centres will send the whole vitrectomy cassette for further testing.

The steps of diagnostic vitrectomy are summarized in Figure 4.

Recently, DeBoer et al. [37] introduced an alternative, novel in-clinic vitreous biopsy technique that utilizes a single peel pack 25-gauge vitrectomy cutter, eliminating the requirement for a surgical console or complex setup. This technique involves trimming the cutter’s drive line and connecting it to a 10-mL syringe for manual activation. At the same time, a 1 mL syringe is attached to the aspiration line, providing a controlled suction. After placing the trocar-assisted cannula via the pars plana, the cutter is directed into the vitreous cavity, and cutting is conducted manually by coordinating syringe manipulation. As soon as the sample is obtained, intravitreal antimicrobials can be administered—if necessary—and the cannula is removed. The authors provide supporting evidence that this technique is simplified, console-free, and efficient, with potentially higher diagnostic yield than traditional needle-based biopsies.

One limitation of the conventional diagnostic vitrectomy is that the sample becomes diluted in balanced salt solution. To overcome this issue, the vitreous fluid can be manually aspirated with a syringe attached to the aspiration line of the vitreous cutter. The vitrectomy machine’s automated air pump is then used to replace the aspirated vitreous with air [34].

### 4.3. Chorioretinal Biopsy

If the inflammatory process is mainly affecting the neurosensory retina or RPE and the diagnosis is still indeterminate even after analysis of the vitreous sample, then chorioretinal biopsy could be considered. This approach can be helpful in detecting aetiologies such as tuberculosis, sarcoidosis, cytomegalovirus, and lymphoma, especially in cases with diagnostic uncertainty [3,7,29,38,39]. Considering that chorioretinal biopsies provide a larger amount of tissue compared to vitreous sampling, they can facilitate a more precise classification of the lesion, as well as differentiation via immunohistochemical methods [3]. However, considering the morbidity related to the procedure, biopsies are usually the last resort for establishing a diagnosis [7,38].

The first description of the ab interno chorioretinal biopsy technique is credited to Peyman [40]. Over the years, variations of this approach— such as choroidal biopsy, subretinal aspiration, endoretinal biopsy, and ab externo chorioretinal biopsy— have been reported in the management of several intraocular disorders (e.g., subretinal fibrosis, ocular sarcoidosis, ARN, PVRL, TB-related uveitis, Candida endophthalmitis, and HTLV-1–associated leukaemia) [29,41,42,43,44,45]. The primary indication for chorioretinal biopsy is suspected PVRL. Although vitrectomy is more commonly employed for diagnostic purposes in PVRL, vitreous sampling often yields false-negative results, even after repeated procedures [34].

The key surgical considerations for performing a chorioretinal biopsy are summarized in Table 1 [46,47,48,49,50,51].

### 4.4. Laboratory Diagnostic Techniques

**Figure 5:**
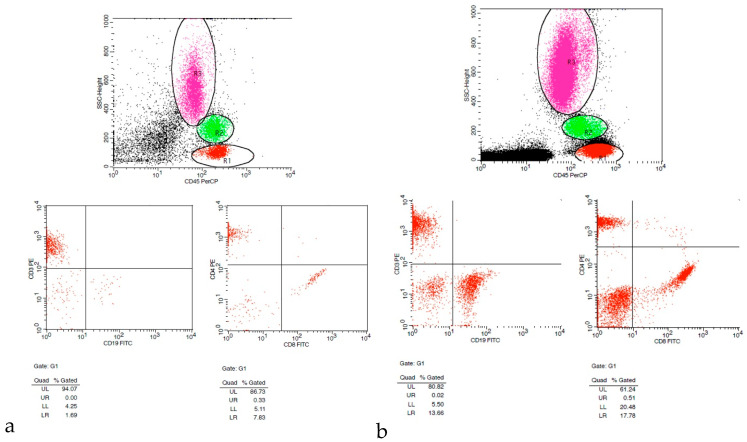
Flow cytometric analysis of vitreous (**a**) and peripheral blood (**b**) immune cells in a female patient with unilateral tuberculous panuveitis. Flow cytometry of vitreous fluid (**a**) revealed CD19^+^ B cells (94%), CD16^+^56^+^ natural killer cells (4%), CD3^+^CD4^+^ helper T cells (87%), and CD3^+^CD8^+^ cytotoxic T cells (7%), resulting in a CD4^+^/CD8^+^ ratio of 12.5. Differential analysis showed 25% lymphocytes, 15% monocytes, and 60% polymorphonuclear cells. In contrast, peripheral blood (**b**) analysis demonstrated CD19^+^ B cells (82%), CD16^+^56^+^ NK cells (5%), CD3^+^CD4^+^ T cells (63%), and CD3^+^CD8^+^ T cells (18%), with a CD4^+^/CD8^+^ ratio of 3.8. These findings illustrate a marked enrichment of CD4^+^ T cells in the vitreous compared with the peripheral circulation, reflecting a local Th1-skewed, cell-mediated immune response characteristic of tuberculous uveitis. CD4^+^ T cells likely drive macrophage activation to control *Mycobacterium tuberculosis* within the eye, while monocytes contribute to granulomatous activity. The high proportion of polymorphonuclear cells in the vitreous suggests concurrent acute inflammation. In contrast, the blood profile shows a more balanced CD4^+^/CD8^+^ ratio, highlighting that the immune response within the eye is locally amplified and compartmentalized. Image courtesy of Professor Chris Kalogeropoulos and Dr. Georgios Vartholomatos, University Hospital of Ioannina, Greece. R1, R2, and R3 are shown in different colours simply to distinguish different gated cell populations on the flow-cytometry plot.

The diagnostic approach to uveitis is often challenging. In some cases, surgical intervention can contribute to diagnostic and therapeutic orientation [3]. Although traditional histopathological and microbiological investigations are fundamental in specific uveitic entities (e.g., syphilitic uveitis, Lyme disease-associated uveitis), they are not always enough for establishing a definitive diagnosis. For example, in toxoplasmic chorioretinitis and herpetic eye disease, making an accurate diagnosis can be more complex due to widespread seropositivity in healthy individuals. Thus, further diagnostic methods, such as the Goldmann–Witmer coefficient (GWC), microbial culture, cytology, flow cytometry [Figure 5], molecular testing [e.g., real-time polymerase chain reaction (PCR) or metagenomics], and cytokine profiling can improve diagnostic accuracy [3]. In general, surgical sampling of aqueous, vitreous, or chorioretinal tissue is indicated when there is suspicion of infection, concern for malignancy, or failure of empiric medical therapy to control intraocular inflammation. Employing one or more tests depends on the suspected underlying pathology [3]. Celiker et al. [5] reported an overall diagnostic yield from vitreous sampling of 41.7%, detecting infectious aetiologies such as fungal and bacterial endophthalmitis, cytomegalovirus retinitis, and non-infectious causes such as PVRL. *Cytological* assessment can define the vitreous cell phenotypes. For instance, the diagnosis of PVRL relies largely on the detection of large B-cell blasts and atypical lymphocytes. Likewise, cytology can contribute to the diagnosis of sarcoidosis by recognizing multinucleated giant cells, along with lymphocytes and epithelioid cells [7,52]. On the other hand, in some cases, *histopathology* has demonstrated greater sensitivity compared to immunohistochemistry and molecular analysis. For example, in ocular toxocariasis, histological examination may demonstrate membranes with granulomatous tissue, whereas samples from ocular tuberculosis often reveal necrotizing granulomatous inflammation and giant cells [7,13]. Van Ginderdeuren et al. [53] proposed a standardized protocol for vitreous biopsy sampling and processing using the fully automated Cellient cell block system, demonstrating that diagnostically adequate material could be obtained in more than 90% of cases. This approach allowed reliable morphological evaluation and immunohistochemical staining, despite the low cellularity and viscous nature of vitreous samples, facilitating differentiation between inflammatory, infectious, and malignant intraocular conditions. When suspecting an infectious cause, *microbial cultures* are performed together with Gram staining and antibiotic sensitivity testing. There are various culture media that can be used depending on the suspected organism: blood agar, MacConkey agar, Brucella agar for bacteria; Sabouraud dextrose agar for fungi and yeasts. However, viral cultures for vitreous samples tend to have a relatively low sensitivity. This is most likely attributed to the relatively low viral load or the presence of neutralizing antibodies [7]. *Molecular analysis*, especially PCR, can be extremely helpful in the differential diagnosis of masquerade syndromes, such as PVRL, and also for detecting infectious agents [herpesviruses (HSV, VZV, CMV), *Toxoplasma gondii*, *Mycobacterium tuberculosis*, *and other bacteria*] [54,55]. Particularly in PVRL, PCR can establish a diagnosis and also evaluate prognostic biomarkers [56,57]. In atypical cases of uveitis, PCR can contribute to the detection of pathogens such as *Propionibacterium acnes* or *Mycobacterium tuberculosis*. It has been suggested that incorporating PCR into the standard microbiological analysis can increase sensitivity from 48% to more than 80% [3,58,59,60]. Particularly in ocular tuberculosis, drug resistance can make the management of the disease very challenging. Early recognition of multidrug resistance (especially to rifampicin and isoniazid) can contribute to controlling the inflammatory activity and, therefore, preventing potential vision-threatening complications. Although traditional drug susceptibility techniques are slow, novel molecular assays can provide faster results. The WHO-endorsed Xpert MTB/RIF assay detects *Mycobacterium tuberculosis* DNA and rifampicin resistance within two hours using a cartridge-based PCR system targeting the rpoB gene [61,62]. The GenoType MTBDRplus test offers additional detection of katG and inhA mutations, enabling the detection of both high- and low-level isoniazid resistance in about five hours [63,64]. Flow *cytometry* is another valuable diagnostic tool in PVRL diagnosis, as it can perform analysis of cell surface markers to define monoclonal B-cell populations. When combined with cytological examination, diagnostic accuracy for lymphoma improves significantly [56,57,65]. It is crucial to highlight that, prior to performing a vitreous biopsy in cases suspected of PVRL, it is recommended to discontinue the use of topical or oral steroids for at least two weeks to allow greater vitreous cellular infiltration and subsequently more accurate results. Moreover, vitreous samples must be promptly delivered to the laboratory, as lymphoma cells rapidly undergo morphological degradation. Therefore, the laboratory should be notified in advance to ensure timely processing of the specimen [56,57].

Apart from diagnostic orientation in infectious aetiologies [66], cytokine and chemokine profiling are also essential in the diagnosis of B-cell malignancies. PVRL is often associated with elevated interleukin-10 (IL-10), an immunosuppressive cytokine, as well as high levels of pro-inflammatory IL-6. An IL-10:IL-6 ratio greater than 1 is suggestive of PVRL. These biomarkers can help confirm the diagnosis and monitor treatment response [56,57]. Apart from the diagnosis of PVRL, flow cytometric analysis of the intraocular fluids can provide crucial information for the diagnosis of uveitis by evaluating the CD4/CD8 ratio and the distribution of inflammatory cells [3]. A CD4/CD8 ratio <1 is suggestive of a viral aetiology, whereas a ratio >3 is more consistent with autoimmune or granulomatous conditions such as sarcoidosis or tuberculosis. The proportion of polymorphonuclear leukocytes (PMNs) offers additional diagnostic value, with >70% PMNs indicating a likely septic infection that warrants culture, and ≤30% PMNs favouring a non-septic autoimmune process, as seen in diseases such as ankylosing spondylitis with severe iritis or Adamantiades–Behçet’s disease [3].

### 4.5. Surgical Outcomes and Complications of Diagnostic PPV

According to a study by Zhao et al. [32], the success rate of diagnostic PPV in studies reported up to 2017 was 44%, yielding a definitive diagnosis in cases with infectious uveitis (69%), lymphoma (23%), and metastatic carcinoma (4%). Among the infectious cases, the most common pathogens were viruses, followed by bacteria, *Toxocara canis*, *Toxoplasma gondii*, *Mycobacterium tuberculosis*, and fungi. The reported incidence of postoperative cataract and retinal detachment was 19% and 5%, respectively. The rate of secondary vitrectomy was 10%, while the postoperative visual improvement was 46%. The results of the diagnostic vitrectomy changed the diagnostic orientation and postoperative therapeutic approach in 20% of cases.

## 5. The Therapeutic Applications of Vitrectomy in Uveitis

**Figure 6:**
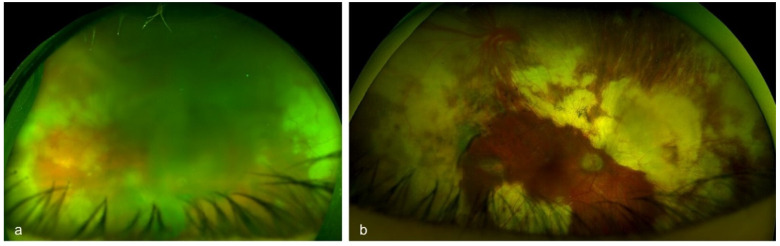
(**a**) Optos widefield colour photography of a 56-year-old immunocompetent male demonstrating prominent vitritis in the right eye, with foci of retinal necrosis showing discrete borders located in the peripheral retina, accompanied by haemorrhagic features and possible vasculopathy—findings suggestive of acute retinal necrosis (ARN). Despite aggressive treatment with intravitreal and systemic antivirals, the patient developed a retinal detachment (RD), which was managed with pars plana vitrectomy. (**b**) Retinal appearance at the patient’s last follow-up, showing a flat retina, with extensive areas of necrosis and retinal thinning secondary to ARN and the associated RD. Image courtesy of the Vitreoretinal team at Stoke Mandeville Hospital, UK.

**Figure 7:**
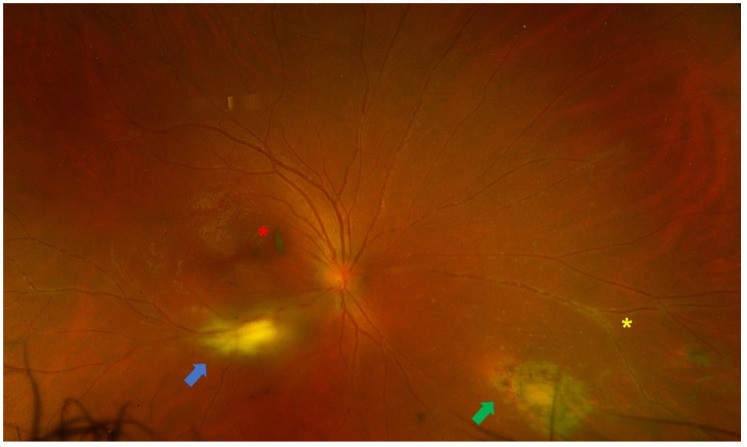
Optos widefield colour photography of a 23-year-old male with unilateral (right eye) toxoplasmic retinochoroiditis. The green arrow indicates an old, scarred, and atrophic inactive lesion in the mid-peripheral inferonasal retina, while residual features of vasculopathy can be seen adjacent to this area (yellow asterisk). The blue arrow highlights an active new lesion along the inferotemporal arcade with overlying vitritis. A few vitreous floaters are also visible (red asterisk). Image courtesy of Mr. Farid Afshar, Consultant Ophthalmologist, University Hospital Southampton, UK.

The indications for performing a therapeutic PPV in uveitic eyes can be categorized based on both anatomical or aetiological classification and surgical considerations [Table 2]. According to previous systematic reviews, from an anatomical standpoint, intermediate uveitis and panuveitis have been reported as the most frequent indications for therapeutic PPV [6,8]. In both cases, the decision to proceed with PPV is mostly guided by the degree of vitritis. Notably, data from the Systemic Immunosuppressive Therapy for Eye Disease (SITE) Cohort Study suggested that PPV was among the factors linked to disease remission in intermediate uveitis [67]. On the other hand, it can be argued that cases with inflammation confined entirely to the choroid are less likely to benefit from therapeutic PPV due to minimal vitreous involvement. Considering the underlying aetiology, therapeutic PPV has been implemented in both infectious and non-infectious uveitic entities [Table 3]. Among non-infectious causes, Adamantiades–Behçet’s disease and juvenile idiopathic arthritis (JIA) were the most common indications, whereas acute retinal necrosis (ARN) [Figure 6] and ocular toxoplasmosis [Figure 7] were the predominant infectious pathologies [8].

### 5.1. Controlling Inflammatory Activity Through Vitrectomy

Conventional treatment of uveitis relies on topical and systemic corticosteroids, immunomodulatory agents (e.g., mycophenolate mofetil, methotrexate, azathioprine, cyclosporine), and biologics (e.g., adalimumab, infliximab) [3]. Kaplan [23,68] first proposed that vitreous removal could augment the penetration and efficacy of inhibitory factors within the vitreous cavity, improving intraocular inflammatory control. Early studies by Algvere et al. [16], Leuenberger [26], and Freyler [27] supported that vitrectomy reduced inflammatory activity, recurrences, and dependence on anti-inflammatory or immunosuppressive treatment.

Later studies reported that removal of the sponge-like vitreous gel facilitates clearance of pro-inflammatory immune cells [69,70,71], immunogenic antigens (e.g., microbial products and type II collagen) [72], and inflammatory mediators such as interleukins, cytokines, and growth factors. Their elimination may reduce antigen presentation and strengthen systemic treatment efficacy [73,74,75,76]. Additional mechanisms include improved drug penetration, altered intraocular oxygen gradients, accelerated inflammatory cell clearance, and reduced secondary immune activation. In eyes undergoing combined lensectomy, formation of a unicameral eye may further enhance diffusion of anti-inflammatory mediators (e.g., TGF-β) from the anterior to posterior chamber, potentially promoting regulatory T-cell activation and inflammation resolution [77]. These observations align with reports showing favourable outcomes following combined phacoemulsification and PPV in chronic uveitis [78,79].

Eckardt and Backsulin [80] assessed 42 eyes with intermediate uveitis that underwent vitrectomy with or without lensectomy, with a mean follow-up of 29 months. Visual acuity improved in 64% of eyes, remained stable in 19%, and worsened in 16%. Chronic inflammation subsided in 88% of cases, with marked reduction in anterior chamber activity and decreased need for treatment with steroids. Visual outcomes may have been more favourable with combined surgery.

Heiligenhaus et al. [81] retrospectively analysed PPV in complex chronic uveitis, including cases with combined lensectomy. After a mean follow-up of 45 months, visual acuity improved in 82.8% of eyes, with a substantial reduction in inflammatory severity, recurrences, and corticosteroid use.

In a randomized prospective study, Tranos et al. [10] compared vitrectomy with medical therapy in CMO secondary to chronic intermediate or posterior uveitis. At 6 months, 50% of surgically treated eyes improved by at least two lines, compared with 18% in the medical group, while angiographic CMO resolution was higher in the surgical group (33% vs. 14%). Similarly, a prospective trial comparing PPV with systemic immunomodulatory therapy in refractory intermediate uveitis revealed visual improvement in both groups, with PPV being more effective for CMO and medical therapy for diffuse macular oedema [11]. These findings underscore the importance of adjusting treatment to the macular oedema subtype.

Apart from vitreous inflammation and CMO reduction [82], PPV may aid the resolution of chorioretinal inflammation. In TB-associated uveitis, adjunctive PPV resulted in substantially higher rates of lesion resolution and greater visual improvement compared with fellow eyes receiving medical therapy alone [83]. This supports the hypothesis that removal of activated T-cells from the vitreous suppresses retinal and choroidal inflammation, analogous to surgical interventions used in refractory inflammatory arthritis [84]. However, certain uveitic entities linked to systemic inflammatory disease, such as Adamantiades–Behçet’s disease, may show less favourable long-term outcomes after surgery [85]. Importantly, effective perioperative inflammatory control appears critical, as demonstrated by improved outcomes with perioperative infliximab therapy in these patients [86].

Overall, many studies advocate vitrectomy as a therapeutic approach to reducing antigenic load and inflammatory mediators in uveitis, while others support prioritizing medical therapy and reserving PPV for refractory cases due to surgical risks. Future studies comparing or integrating medical and surgical strategies may better define the anti-inflammatory role of vitrectomy across uveitis subtypes.

#### 5.1.1. Cystoid Macular Oedema

CMO is one of the most common complications of uveitis, linked to a substantial reduction in visual acuity in over 50% of patients [3]. Despite aggressive treatment, CMO may persist, especially in eyes with chronic uveitis, even when intraocular inflammatory activity is sufficiently controlled [87,88]. The pathogenesis of uveitic CMO has not yet been fully elucidated, but it is believed to involve disruption of the inner and outer blood–retinal barriers, accompanied by migration of inflammatory cells. Removal of inflammatory mediators from the vitreous cavity may contribute to CMO resolution by reducing antigen presentation and enhancing the efficacy of systemic treatment. Mechanical factors may also be involved, as persistent posterior vitreous adhesion is correlated with a higher incidence and more refractory course of CMO compared with complete vitreoretinal separation [74,75,89]. This observation indicates that relieving macular traction (e.g., adherent posterior vitreous or ERM) may promote CMO resolution.

Management of uveitis-associated CMO includes corticosteroids, periocular or intravitreal triamcinolone acetonide, fluocinolone acetonide, dexamethasone implants, anti-VEGF agents, and immunomodulatory therapies. Steroid-sparing agents include biologics (adalimumab, infliximab, tocilizumab), interferon-α, mycophenolate mofetil, and methotrexate [3,87,90,91,92].

A retrospective study of 42 eyes with CMO secondary to intermediate uveitis unresponsive to medical therapy showed that PPV resulted in complete resolution of CMO in 42.8% of eyes and partial improvement in 16.7%, while no change was recorded in 30.9% [93]. Visual acuity ameliorated by at least two lines in 50% of eyes. Although long-term outcomes were slightly reduced due to secondary complications, most patients reduced or discontinued systemic therapy. These findings support PPV as a therapeutic option when medical treatment fails. Likewise, a more recent study demonstrated a reduction in CMO prevalence from 22.6% preoperatively to 12.9% postoperatively, along with decreased systemic immunosuppression in approximately half of patients [5].

Another retrospective study of 19 patients with refractory uveitic CMO evaluated the role of PPV combined with intraoperative intravitreal triamcinolone acetonide. At 6 weeks, CMO improved in 58% of eyes; at 12 months, 44% showed further improvement, and 12% worsened. While PPV with intravitreal triamcinolone may benefit persistent CMO, the effect can be transient and is often associated with ocular hypertension and cataract formation [94].

A review of 44 interventional case series published between 1981 and 2005 assessed PPV in uveitis, including 1575 patients (1762 eyes) [6]. Intermediate uveitis was the most common type of uveitis, frequently associated with CMO and cataract. Among studies reporting visual outcomes, 68% of eyes improved, 20% remained stable, and 12% worsened. The proportion of eyes with CMO decreased from a median of 36% preoperatively to 18% postoperatively, and several studies reported a reduction in systemic therapy. Overall evidence (CII-3) suggested that PPV may improve vision, reduce inflammation, and improve CMO.

A subsequent systematic review of 34 studies published between 2005 and 2014 included 627 patients (708 eyes) [8]. The majority of them were retrospective case series, with low-to-moderate evidence quality (median SIGN grade 3, Oxford grade 4). After PPV, visual acuity improved in 69% of eyes, remained stable in 18%, and worsened in 13%. The prevalence of CMO decreased from 52% to 37%, while the use of oral steroids and other immunosuppressants declined substantially. However, heterogeneity in outcomes and limited prospective data were recorded.

Overall, the role of PPV in uveitis-related CMO remains controversial. Although there is a lack of randomized trials, several studies show favourable outcomes in refractory cases. PPV may therefore be considered for treatment-resistant CMO. Timing is pivotal, as prolonged oedema may cause irreversible macular damage, ERM formation, or macular holes. Considering the heterogeneity of uveitis aetiologies, surgical decisions should be individualized and reserved for selected cases, with outcomes appearing more favourable in certain subtypes (e.g., intermediate uveitis) [1,2]. High-quality controlled studies are needed to better delineate the role of PPV alongside medical therapy [21].

#### 5.1.2. Epiretinal Membranes

Most of the existing studies on ERMs focus mainly on idiopathic ERM, which is primarily related to aging and posterior vitreous detachment (PVD) [95]. In contrast, the estimated incidence of ERM in patients with uveitis, with or without CMO, ranges from 12.6% to 69% [3,96,97,98,99]. According to the SUN classification, the prevalence of ERM was 28.1% in anterior uveitis, 57.0% in intermediate uveitis, and 43.5% in posterior or panuveitis [98]. Although ERM represents a frequent complication in uveitic eyes, the collection of comprehensive data on patient demographics, diagnosis, and disease management is challenging due to the limited number of affected cases [100]. Several studies have investigated the assessment and surgical management of ERMs in patients with uveitis [101,102,103,104,105,106,107,108]. However, their findings may not fully reflect the pragmatic incidence of ERMs related to intraocular inflammation.

Despite being clinically similar to idiopathic ERMs, the pathogenesis of uveitis-associated ERMs remains unclear. Histologically, they are distinguished by the abundance of inflammatory cells and absence of RPE cells [109,110]. However, in some rare cases, inflammatory ERM can resolve spontaneously [111], and surgical removal remains the only definitive treatment, with PPV becoming less invasive due to recent technological advancements [109]. The main indication for surgery is progressive structural or functional decline, ideally after a good control of the inflammation has been achieved [21,109,112]. In general, uveitic ERMs are linked with worse visual outcomes compared to idiopathic ERMs. This could be attributed to a different pathogenetic mechanism or simply because of the higher ophthalmic morbidity of uveitis [113,114]. However, several studies on PPV with ERM peeling show significant improvements in central retinal thickness (CRT) and best-corrected visual acuity (BCVA), with low rates of worsening vision and CMO reduction [103,104,115]. Table 4 summarizes the outcomes of studies evaluating the role of PPV in eyes with ERM secondary to uveitis [100,103,104,106,115,116,117,118]. For a more detailed approach on the clinical features, diagnostic work-up, and management of uveitic ERMs, the reader is referred to our previously published work [109].

#### 5.1.3. Macular Holes

Macular holes (MHs) constitute a relatively rare but still vision-threatening complication of uveitis. The evidence in the current literature regarding their occurrence in uveitis remains limited, and their pathogenesis is not fully understood. In a large study of 413 eyes with uveitis, the authors reported an estimated incidence of 2.5% [113].

Soliman et al. [119] conducted a systematic review to investigate the outcomes of MH medical or surgical treatment (PPV) in eyes with uveitis. The study included a total of 86 eyes across 27 published papers. The mean age of patients was 46.6 ± 16.8 years, with 60.5% male predominance. Adamantiades–Behçet’s disease (34.6%) and toxoplasmosis (19.7%) were the most common aetiologies, and posterior uveitis (59.3%) and panuveitis (35.2%) were the most prominent anatomical locations. Baseline mean LogMAR visual acuity was 1.1 ± 0.5. Conservative medical management was employed in 34.9% of eyes, while 65.1% underwent PPV. Overall, mean LogMAR vision improved from 1.1 ± 0.5 to 0.7 ± 0.5 post-treatment. MH closure occurred in 40% of eyes receiving medical therapy and 87.5% of eyes after PPV, with visual improvement observed in 83.9% of eyes achieving successful closure. These results suggest that good perioperative control of the inflammation may contribute to MH closure and should precede surgery, while surgical intervention is associated with favourable anatomical and functional outcomes [119].

MH formation in uveitis is a result of various mechanisms, including inflammatory damage to the inner retinal layers, recurrent CMO, ERM, and traction from an abnormal hyaloid membrane. It is believed that the underlying processes in idiopathic MH overlap with those of uveitic MH; however, specific clinical features are suggestive of a different pathophysiology. Notably, uveitic MH tends to occur in younger patients (third to fourth decade), whereas idiopathic MH is typically observed in individuals during the sixth to seventh decade of life. Relevant studies report that focal retinitis involving the macula or parafoveal region may progress to necrosis, increasing the risk of full-thickness MH formation [120,121].

PPV with ERM peeling was initially reported by Kelly & Wendel in 1991 as an effective approach for repairing idiopathic MH [122], and it has since become a standard procedure, with favourable visual and anatomical outcomes. By contrast, the surgical management of MH associated with uveitis tends to be more challenging, whereas the anatomical closure can be variable and not always lead to improved vision. This may be related to various parameters such as the chronic effect of inflammation, recurrent CMO, RPE atrophy, and choroidal ischaemia [123,124,125,126].

Likewise, Kahloun et al. [127] evaluated 120 eyes from 65 Adamantiades–Behçet’s patients, identifying full-thickness MH in 3 eyes (2.5%). Two of these patients underwent PPV with ILM peeling and gas tamponade, with MH closure and visual improvement achieved in 1 case.

Table 5 summarizes the clinical characteristics, surgical techniques, and anatomical and visual outcomes of PPV for macular holes associated with uveitis and other inflammatory aetiologies reported in the literature [128,129,130,131,132].

Interestingly, some reports describe successful MH closure solely with medical treatment. A relevant study described 3 cases with rapid resolution and good visual outcomes after achieving a satisfying control of the posterior segment inflammation [133].

Evidence from the current literature suggests that some types of uveitis, such as Adamantiades–Behçet’s disease or cases with macular inflammation and focal necrosis, may present an increased risk of MH formation and indicate a more individualized approach in order to define and manage the associated pathogenetic factors, such as chronic CMO, ERM, or tractional forces. The reports discussed above describe successful closure after both medical and surgical treatment, suggesting that uveitic MHs are not a uniform pathology with a standard therapeutic approach. Future research is needed to understand the exact mechanisms of MH formation in various types of uveitis, along with subgroup analysis, and define which eyes will benefit most from surgery and which surgical techniques are optimal. Until then, it is advised that medical therapy should be utilized prior to surgical intervention, considering the unpredictable visual outcomes of MH surgery in uveitis [21].

#### 5.1.4. Retinal Detachment

The reported incidence of rhegmatogenous RD (RRD) among individuals with uveitis varies between 1.7% and 3.1%, a prevalence significantly higher than that observed in non-uveitic populations (18 per 100,000). Posterior uveitis and infectious aetiologies—especially viral infections causing retinitis (e.g., Herpes Simplex virus, Varicella Zoster virus, and Cytomegalovirus)— have been linked to a higher risk of RRD compared to other causes of uveitis [134,135,136,137].

The highest incidence of non-uveitic RRD is recorded in the seventh decade of life and is typically attributed to the age-related increase in PVD. It has been reported that the mean age of uveitis-related RRD is approximately 40 years, significantly lower than compared to non-uveitic populations. A possible explanation is that PVD develops earlier in life in uveitic eyes. It has been hypothesized that intraocular inflammation may promote crosslink formation within vitreous collagen, leading to subsequent liquefaction and eventually PVD [89]. Further pathological processes associated with uveitis, such as retinal necrosis, vitreous contraction, fibrosis, and tractional forces, may further predispose to retinal breaks and RRD [138,139,140].

Early reports described favourable anatomic outcomes in eyes with RRD secondary to inflammation, with a reattachment rate up to 91%, but limited functional recovery [135]. Later studies on acute retinal necrosis and HIV-associated retinitis reported high rates of RRD (up to 85%) and poor visual outcomes, usually due to recurrent retinitis, optic atrophy, HIV-related microvasculopathy, and cataract [141,142]. A variety of surgical techniques have been described, including scleral buckling, prophylactic laser, and cryotherapy, but these often failed in the necrotic retina. Vitrectomy with silicone oil tamponade emerged as the most effective and promising approach, due to the long-lasting tamponade of multiple and posterior retinal breaks [142,143,144,145]. Iannetti et al. [146] reported long-term outcomes of PPV for RD associated with acute retinal necrosis (ARN). Retinal reattachment was achieved in 91% of cases after 23-gauge PPV with silicone oil tamponade and systemic antiviral treatment. Despite the high anatomic success, final visual recovery was limited, especially in eyes with macula-off RD or involvement of the optic nerve, underlining the significance of early diagnosis and prompt management of ARN and ARN-related RD. Some case series support that prophylactic laser photocoagulation may reduce the risk of RD. However, there is not enough evidence to prove its effectiveness in preventing RD [147,148]. It has been proposed that early vitrectomy in ARN can contribute to the removal of inflammatory mediators and reduction in vitreoretinal traction, and can be considered as a preventive measure against RD [149,150]. A relevant systematic review and meta-analysis [150] highlighted that prophylactic vitrectomy was superior to standard antiviral treatment in reducing the risk of RRD (*p* < 0.001, OR 0.27, 95% CI 0.16–0.46). Nevertheless, the efficacy of prophylactic vitrectomy has not been fully elucidated yet, and further studies are required [148]. The management of RD secondary to ARN poses various challenges, such as a high incidence of proliferative vitreoretinopathy (PVR), multiple retinal breaks in a thin, atrophic retina, and the coexistence of ischaemic optic neuropathy. Before the emergence of vitrectomy, surgical outcomes were generally poor, with anatomical success rates of around 22% [151]. The advancements in surgical techniques and equipment (e.g., the use of vitrectomy combined with silicone oil tamponade) have led to improved anatomical success rates ranging from 78% to 100% [152,153]. Furthermore, concurrent antiviral and anti-inflammatory therapy contributed to better clinical results. According to a retrospective study by Dave et al. [154], a delayed onset of RRD following ARN seems to be associated with a favorable prognosis, whereas early administration of oral valacyclovir and systemic steroids prior to the development of RRD is linked with improved visual outcomes.

Surgical management of the vitreoretinal complications associated with Adamantiades–Behçet’s disease has shown favourable outcomes. Dabour et al. [155] reported 7 cases with RRD for whom retinal reattachment was successfully achieved; scleral buckling was found effective in simpler cases, whereas PPV was performed in more complex eyes. Similarly, long-term data in the study of Mesquida et al. [156] demonstrated that PPV is effective for a wide spectrum of vitreoretinal disorders—such as vitreous haemorrhage, macular holes, and RD—in this group of patients. They showed that PPV led to improved or stabilized vision in nearly half of the cases, reduced dependence on immunosuppressive treatment, and minimal postoperative inflammation.

Comparative data demonstrate inferior outcomes in uveitic than in non-uveitic RRD. A relevant study described lower single-operation success (59% vs. 82%) and poorer final vision in uveitic eyes, especially in those with panuveitis and infectious aetiologies [136]. Likewise, De Hoog et al. [140] recorded an RRD prevalence of 3.1% among uveitis patients. There was a significant correlation with posterior uveitis and infectious causes, with a final reattachment rate of 83% and a mean visual acuity of 20/125; 41% of eyes had VA <20/200. A more recent study [157] evaluated 71 eyes with RRD secondary to intraocular inflammation in order to recognize factors affecting surgical outcomes. The majority of cases were attributed to posterior uveitis or panuveitis, and predominantly of infectious aetiology. Primary reattachment was reported in 74.6% of eyes after a single surgery, while success reached 100% when scleral buckling was used alone or combined with vitrectomy. Better baseline visual acuity (≥20/400) was associated with greater visual gain, while the absence of early post-op recurrence was linked with long-term anatomical success. According to their results, RRD was associated with a favorable anatomical prognosis, whereas scleral buckling may ameliorate surgical outcomes in selected cases.

PPV has been enhanced by recent advances in microincision vitrectomy surgery (MIVS), which offers shorter surgery, less discomfort, faster recovery, and reduced reliance on systemic corticosteroids. Several parameters, such as modern 25-gauge and 27-gauge systems, improved instruments, lighting, and cutters, have contributed to its safety and efficacy [21,106,123,158,159,160,161]. Moharana et al. [152,162] demonstrated a 94% reattachment rate with significant functional improvement in patients with RRD secondary to viral retinitis. Using encircling scleral bands during PPV remains debatable, though there is some evidence favouring their use in complex cases with traction or proliferative vitreoretinopathy (PVR) [152,163].

Complications such as hypotony or PVR remain critical prognostic determinants. Hypotony may arise as a result of damage to the ciliary body [164,165,166,167], whereas incomplete PVD and increased levels of ICAM-1 have been associated with PVR development [136,168,169,170,171]. Combined pharmacologic approaches, such as steroids, methotrexate, anti-VEGF, 5-fluorouracil, low-molecular-weight heparin (LMWH), and daunorubicin, have shown variable results [172,173,174,175,176,177,178,179]. Finally, data from experimental studies suggested that PDGFRα signalling may represent a novel therapeutic target [180,181,182].

Despite improvements in PPV, the overall quality of evidence for uveitis remains limited. While surgery can address structural complications, visual outcomes can be variable. It appears that RRD in uveitic eyes poses a higher risk of re-detachment and poor visual prognosis, with up to 71% of patients ending with VA < 20/200 and 1% with no perception of light [136]. Optimal perioperative inflammatory control and individualized surgical strategies remain pivotal, but further studies are required to establish effective practices and treatments.

#### 5.1.5. Vitreous Opacities

A recent report by Alisi et al. [183] showed that PPV may offer considerable functional improvement and anti-inflammatory effect in patients with persistent vitreous opacities associated with infectious or non-infectious uveitis that does not respond to medical treatment. According to their results, there was a notable reduction in the inflammation of the anterior segment and vitreous haze on the 6-month postoperative follow-up. Visual outcomes were favourable, indicating an improvement of the BCVA in about 72% of the operated eyes. The study reported that preoperative BCVA was a key predictor of postoperative visual improvement (coefficient = 0.60, 95% CI: 0.22–0.98, *p* = 0.003), whereas the underlying aetiology and the lens status had no substantial impact. The recorded postoperative complications included transient intraocular pressure increase in five patients, with two requiring surgical management (i.e., trabeculectomy). Two cases progressed from ERM to tractional RD. Overall, it was suggested that PPV is considered a safe and effective treatment for persistent uveitis-related vitreous opacities. Despite the good visual rehabilitation and the long-lasting inflammatory control, the authors highlighted the importance of meticulous postoperative monitoring to manage any potential complications in a timely manner. The afore-mentioned findings are in agreement with other relevant studies [76,184], which also suggest that PPV can have a beneficial effect on visual function, improve the control of vitreous inflammation, and reduce the need for systemic steroids.

Apart from the improvement in visual acuity, the removal of vitreous opacities from chronic inflammation or haemorrhage can play a pivotal role in providing an adequate visualization of the posterior pole. Retinal neovascularization and vitreous haemorrhage secondary to uveitis are most commonly associated with pathologies such as pars planitis, Eales’ disease, Adamantiades–Behçet’s disease, ocular sarcoidosis, and lupus-related retinal vasculitis. In cases where vitreous haemorrhage is a result of retinal neovascularization, PPV is often combined with endolaser photocoagulation to reduce intraocular VEGF levels associated with peripheral retinal ischaemia [34].

#### 5.1.6. Hypotony Secondary to Cyclitic Membranes

In patients with chronic uveitis, tractional forces exerted on the ciliary body by a cyclitic membrane can lead to hypotony. These membranes may cause detachment of the ciliary body, which can, in turn, lead to decreased aqueous production and increased aqueous outflow. This condition can cause substantial visual deterioration through the development of hypotony maculopathy and choroidal detachment. Uveitic eyes with chronic hypotony are considered pre-phthisical, as progressive anatomical disorganization and globe shrinkage may follow if left untreated. When hypotony does not respond adequately to medical anti-inflammatory therapy, PPV with cyclitic membranectomy can be considered as an alternative option for restoring and maintaining IOP. In these cases, the use of silicone oil is recommended to achieve sufficient tamponade and prevent aqueous misdirection. The removal of silicone oil can be considered as soon as the production of aqueous humour and IOP normalize [34].

#### 5.1.7. Endophthalmitis

PPV may have a therapeutic effect in endophthalmitis cases by removing a considerable reservoir of pathogens from the intraocular milieu. EVS showed that, in post-cataract endophthalmitis, patients with visual acuity of light perception or worse achieved better visual outcomes following vitrectomy compared to those who underwent vitreous tap and injection [185]. The EVS, however, did not include endophthalmitis cases of other aetiology (e.g., trauma, endogenous infection, bleb-related or delayed-onset disease, and post–penetrating keratoplasty). Recent evidence underlines the significance of early and near-complete PPV over limited vitreous removal, as it may lead to better clearance of the infectious component and improved anatomical outcomes. In the context of endophthalmitis, surgical procedures span a wide spectrum, ranging from anterior chamber and vitreous taps or biopsies to therapeutic interventions like vitrectomy, intraocular lens explantation, lens abscess removal, and subretinal abscess drainage [186]. As already highlighted above, advances in microsurgical techniques, wound management, and intraoperative visualization have significantly improved safety and outcomes, though challenges such as hypotony, media haze, and infection control persist [186]. A multicentre retrospective cohort study used data from the American Academy of Ophthalmology IRIS^®^ Registry to evaluate visual outcomes in 1044 patients with endophthalmitis following intravitreal anti-VEGF injections between 2016 and 2020, to compare the efficacy of intravitreal antibiotics alone versus early PPV plus intravitreal antibiotics. After 1:1 Mahalanobis distance matching (n = 218; 109 per group) based on baseline and diagnostic visual acuity, the median logMAR visual acuities were 0.32 at baseline, 0.88 at diagnosis, and 0.57 posttreatment. Interestingly, there was no statistically significant difference in final visual outcomes between the two therapeutic approaches (b = 0.05; *p* = 0.23), including among patients with poorer presenting vision (VA > 1.0 logMAR). These results suggest that both early vitrectomy with antibiotics and antibiotic injection alone yield comparable visual outcomes in post-injection endophthalmitis, supporting clinical flexibility in initial management approaches [187]. Moreover, the results of the EVIAN study [188], a clinical trial comparing early vitrectomy versus intravitreal antibiotics for postoperative exogenous endophthalmitis, are yet to be reported, expecting that more light will be shed in terms of the optimal management of the disease. More recently, Wassef et al. [189] conducted a multicentre retrospective study to compare surgical and medical management of endogenous fungal endophthalitis across 404 patients. They found that eyes treated with vitrectomy, alongside systemic antifungal therapy, showed significantly greater visual improvement than those managed medically alone. Thus, vitrectomy should be strongly considered in cases where the clinical scenario necessitates rapid and targeted management. Ultimately, it is crucial to underline that in endophthalmitis cases, surgical approaches should be guided by the severity of the disease, causative organism, and ocular status in order to achieve optimal anatomical and functional recovery.

#### 5.1.8. Paediatric Uveitis

Studies on PPV in paediatric uveitis demonstrate that it can be a safe and effective therapeutic option for the management of chronic intraocular inflammatory activity and its associated complications when medical treatment is insufficient. The Massachusetts Eye Research and Surgery Institution reported successful uveitis control in 96% of cases, with reduced systemic medication needs, though cataract formation and retinal tears were noted in some eyes [190]. Similarly, long-term outcomes from other centres demonstrated that PPV can substantially improve visual acuity and control inflammation, even in cases refractory to standard immunosuppressive treatment strategies [191,192]. When combined with cataract extraction and intraocular lens implantation, PPV also achieved favourable visual results in children with uveitis and vitreoretinal disorders, though postoperative complications such as glaucoma and CMO were reported [192]. Overall, evidence supports PPV as a useful therapeutic approach in selected cases of paediatric uveitis, especially for resistant cases or those with vitreoretinal involvement.

### 5.2. Surgical Outcomes and Complications of Therapeutic PPV

According to a recent review by Vithalani & Basu [9], therapeutic PPV can lead to improved visual outcome and reduction in various inflammatory indicators (e.g., vitreous haze, resolution of CMO, and reduction in oral corticosteroids or other immunosuppressive drugs). These findings are in agreement with previous reviews [6,8]. However, the results of the included studies face limitations arising from the absence of well-controlled, prospective data. For instance, the improvement of CMO—a key sign of posterior segment inflammation— could result from improved inflammatory control or reduced tractional forces, but it may also have been influenced by other confounding factors, such as dexamethasone implants [193].

Based on current evidence, complications related to therapeutic PPV have not been comprehensively reviewed in a systematic manner. The most frequently reported complications include cataract progression, elevated IOP, postoperative hypotony, CMO, iatrogenic retinal breaks, and retinal detachment. However, it appears that the incidence of iatrogenic retinal breaks was comparable to that recorded in non-uveitic eyes [9,194,159,195].

## 6. Future Perspectives

### 6.1. Understanding the Immunological Background of Uveitis

It is expected that further improvements in the diagnostic and therapeutic applications of PPV may enhance our understanding of the immune mechanisms of uveitis in humans and animals. Although animal models of uveitis have been extensively investigated [196], much remains to be learned about the immune mechanisms underlying human uveitis. The majority of studies so far have focused on exploring the immunological milieu in specific uveitic entities. For instance, Adamantiades–Behçet’s disease is defined by non-granulomatous inflammation and infiltration with perivascular T-lymphocytes and neutrophils [197]. The lymphocytes also exhibit resistance to Fas-mediated apoptosis, which may play a role in sustaining chronic inflammation. On the contrary, Vogt-Koyanagi-Harada syndrome is distinguished by granulomatous inflammation, marked by predominant T-lymphocyte infiltration and a higher CD4+/CD8+ ratio [198]. On the other hand, in TB posterior uveitis, highly pro-inflammatory CD4+ T-cells prevail in the vitreous infiltrate. Many of them are multifunctional and produce multiple cytokines (e.g., IFNγ, TNFα, and IL-17). Notably, these cells respond to both TB antigens and retinal autoantigens. Similar to Adamantiades–Bechet’s disease, the autoreactive T-cells are not only highly pro-inflammatory but also resistant to activation-induced cell death, leading to prolonged intraocular inflammatory activity. It has been suggested that the inflamed vitreous constitutes a “reservoir” of diverse pro-inflammatory immune cells and their cytokines; these are involved in directly mediating inflammation and, particularly in the case of CD4+ helper T-cells, intensifying it by further recruitment of immune cells [69].

### 6.2. Surgical Innovation and Robotic-Assisted Vitreoretinal Surgery

Robotising vitreoretinal procedures has been introduced as a promising and innovative approach to overcome the intrinsic barriers of manual intraocular microsurgery, where paramount precision is mandated to perform safe operations within the compact and fine vitreoretinal milieu [199]. Physiological hand tremor, limited depth perception, and restricted instrument dexterity constitute some of the most critical challenges in vitreoretinal surgery, particularly during membrane peeling, subretinal drug delivery, and retinal vein cannulation, in which errors on the scale of microns may cause permanent retinal damage [200]. Robotic platforms have shown the ability to substantially reduce tremor, enable motion scaling, and increase stability, allowing more accurate and controlled surgical manipulation compared to the human hand alone [201]. Recent advances in robotic systems—including telemanipulator and co-manipulator architectures—have augmented the translation of these novel technologies from pre-clinical models into first-in-human studies. In particular, systems such as the Preceyes Robotic Surgical System and the KU Leuven robotic platform have demonstrated safety, feasibility, and clinical applicability in vitreoretinal surgery [202,203,204]. Integration of robotic platforms with intraoperative imaging modalities, particularly OCT, can further address obstacles regarding visualization and depth perception, leading to improved surgical precision and intraoperative decision-making [205].

This evolution has taken place alongside major developments in conventional vitreoretinal surgery, including minimally invasive surgical techniques that have significantly upgraded outcomes for various vitreoretinal pathologies such as epiretinal membranes, macular holes, and rhegmatogenous retinal detachments [206,194,207,208]. Despite these achievements, complex pathologies such as proliferative diabetic retinopathy (PDR), PVR, retinal vascular occlusions, and retinal degenerative diseases continue to create pivotal diagnostic and therapeutic challenges due to the paramount tenuousness of retinal tissue and the physical constraints of human manual precision [209,210]. At the same time, emerging therapeutic advances—including gene therapy, cell therapy, and regenerative medicine—call for unprecedented accuracy and stability for successful intraocular delivery, which apparently cannot be easily achieved by conventional manual surgery [210,211,212,213]. After decades of growth in industrial and medical robotics, ophthalmology-specific robotic systems aim to offer tremor-free, highly reproducible micromovements adjusted to ophthalmic microsurgery, where accuracy at the scale of microns is crucial [214]. Consequently, a wide range of ophthalmic robotic systems has evolved, stretching from active surgical robots to assistant and observation robots, designed to facilitate compatibility with microscale instruments and rapid response to patient movement [200]. Early clinical applications (e.g., robot-assisted membrane peeling and subretinal injections) have shown that the use of these systems in humans is safe. However, it has been reported that procedure times tend to be longer during the initial phases of clinical adoption [203,204,205].

The OQrimo^®^ system, recently launched and implemented at Kyushu University Hospital, has been shown to effectively stabilize the intraocular endoscope during pars plana vitrectomy, enabling consistent visualization of the peripheral retina without the need for scleral indentation [215]. Notably, no intraoperative or postoperative complications were recorded in the initial clinical cases, supporting the safety and suitability of this system for routine clinical use [215]. It is expected that the convergence of robotic platforms with real-time imaging, artificial intelligence-guided control, and automated instrument handling will further enhance surgical precision, promote standardization of procedure, minimize dependence on individual manual skill, and expand the indications for robotic-assisted interventions in vitreoretinal disorders [216].

### 6.3. Artificial Intelligence

The emergence of artificial intelligence (AI) together with recent technological developments in ophthalmic imaging, surgical visualization, and robotics is gradually reforming the diagnosis and management of uveitic [217] and vitreoretinal pathologies [218,219,220]. Deep learning algorithms have shown high accuracy in detecting and prognosticating several vitreoretinal conditions (e.g., epiretinal membrane, macular hole, retinal detachment, and vitreomacular traction) by using multimodal imaging, such as widefield fundus photography, OCT, and OCT angiography [221,222,223,224,225,226]. Moreover, advances in preoperative imaging (e.g., ultrawidefield imaging, swept-source, and handheld OCT) have significantly improved the examination of peripheral retinal disorders in adults and children [227,228,229]. Intraoperative technologies, including heads-up and head-mounted three-dimensional visualization systems and microscope-integrated intraoperative OCT, enhance surgical ergonomics, visualization, and real-time decision-making [230]. As mentioned above, emerging robotic platforms, often integrated with OCT guidance and AI-based control algorithms, provide impressive precision for delicate manoeuvres, such as subretinal and intravascular drug delivery [200,202,203]. Despite challenges related to limited datasets, imaging artifacts, cost, workflow integration, and ethical considerations [231], the implications of AI, advanced imaging, and robotic surgery appear promising and are expected to improve diagnostic accuracy, surgical planning, and surgical outcomes in the management of vitreoretinal diseases.

### 6.4. Limitations of the Reviewed Studies and Future Directions

The interpretation of the listed references is subject to various limitations inherent to the available evidence in the current literature. First, one of the major limitations is the heterogeneity in study design. Most of the studies evaluating PPV in uveitis are retrospective case series or observational cohorts, while there are only a few prospective or randomized studies. Apparently, this constrains the ability to draw solid conclusions about causality, efficacy, and optimal timing of surgery.

Second, as it has already been underlined, uveitis encompasses a wide range of infectious and non-infectious aetiologies, with anatomical subtypes and variable disease severity. As can be inferred, this creates a significant heterogeneity across studies. Indications for PPV, surgical techniques, and postoperative management vary substantially, posing considerable challenges in making direct comparisons across studies and, therefore, limiting generalizability.

Third, it is obvious that outcome measures are not consistently reported. Visual acuity, inflammatory control, recurrences, and complication rates do not have standardized definitions or measurements. Notably, patient-reported outcomes and quality-of-life measures are infrequently analysed, despite their significance and relevance in chronic inflammatory ophthalmic disease.

Fourth, older studies—particularly those published before the implementation of small-gauge vitrectomy, multimodal imaging, and molecular diagnostics—may not fully depict current diagnostic capabilities or modern surgical approaches. On the contrary, more recent reports often include smaller cohorts of patients or highly selected groups, which may often lead to selection bias.

Finally, limited incorporation of molecular and/or immunological information moderates our understanding of how underlying inflammatory mechanisms affect anatomical and functional outcomes. The majority of studies on diagnostic vitrectomy focus on cytological analysis or pathogen detection without creating adequate correlations with systemic disease activity, cytokine profiles, or predictive biomarkers.

It is recommended that future studies should emphasize prospective, multicentre designs with standardized inclusion/exclusion criteria, well-defined surgical protocols, and clear outcome measures. It is also essential to provide subgroup analyses relevant to the aetiology of uveitis, anatomical classification, and chronicity of the disease in order to detect individuals who are most likely to benefit from PPV. Prolonged follow-up, evaluation of patient-reported outcomes, and incorporation of immunological and molecular profiling may contribute to establishing more accurate predictive biomarkers and optimizing tailor-made therapeutic strategies. Such approaches are required to define the exact role of PPV in the management of uveitis and enhance the evidence-based decision-making in this unique group of patients.

## 7. Conclusions

Since its introduction approximately 5 decades ago, PPV has evolved substantially. Earlier outcomes were restricted due to suboptimal instrumentation, long operating times, and limited surgical expertise, restraining its use to advanced cases of chronic uveitis and typically yielding poor results. With the introduction of small-gauge systems and minimally invasive techniques, PPV has become gradually safer and more efficient, broadening its implementation in both diagnostic and therapeutic procedures. These advances have allowed ophthalmic surgeons to manage complications arising from uveitis more effectively and to perform diagnostic vitrectomies for establishing a diagnosis when paired with the appropriate ancillary laboratory testing.

Despite these improvements, the results of PPV in uveitis remain controversial, and the current evidence is heterogeneous and of variable quality. Several published studies include diverse uveitic aetiologies, inconsistent surgical techniques, and non-standardized reporting of outcomes, which limits comparability and predictive value. Although various structural abnormalities may respond favourably to the surgical treatment, final visual outcomes remain unpredictable, as they are related to multiple parameters, such as the severity of the disease, perioperative inflammatory control, and associated complications.

## Figures and Tables

**Table 1:** Key surgical considerations for chorioretinal biopsy [46,47,48,49,50,51].

Procedure Phase	Key Considerations
Overall approach	Chorioretinal biopsy is most commonly performed using a transvitreal approach
Preferred biopsy site	Superior and nasal retinal regions are generally preferred
If retinal detachment is present	The biopsy should target the junction between attached and detached retina
If retina is attached	Subretinal saline is injected using a 41-gauge cannula to create a small bleb
Purpose of bleb formation	Creates a safety margin above the RPE before tissue sampling
Suspected retinitis	Biopsy should include the advancing edge of the lesion, where pathogen activity is highest
Extent of vitrectomy	Only minimal vitrectomy is required to safely access the target tissue
Posterior vitreous detachment	Induction should be avoided to reduce the risk of retinal tears
Bleeding control	Temporary elevation of intraocular pressure can reduce haemorrhage risk
Cutter settings	Low cut rate combined with high suction is recommended
Tissue acquisition	Manual aspiration with a syringe while gently rotating the cutter
Retinal stabilization	Endolaser or endodiathermy may be applied around the biopsy site
Endotamponade	Long-acting gas or silicone oil can be used
Postoperative care	Postoperative posturing is important for effective retinal tamponade
Specimen processing	Samples should be divided for cultures, histopathology, PCR, and immunological analysis
Patient counselling	Patients should be informed about the risk of retinal detachment and possible false-negative results

**Table 2:** Therapeutic pars plana vitrectomy in uveitis [9,14,21].

Main Indications	
Aetiological/Anatomical Indications	Non-infectious: Intermediate uveitis/pars planitis Paediatric uveitis (including JIA-associated uveitis) Sarcoidosis Adamantiades–Behçet’s disease HLA-B27 associated uveitis Vogt–Koyanagi–Harada disease Birdshot chorioretinopathy
	Infectious: Infectious endophthalmitis Viral retinitis Ocular toxoplasmosis Ocular tuberculosis Fuchs heterochromic iridocyclitis Undiagnosed infectious uveitis
Surgical indications	Stand-alone surgery: Lack of sufficient improvement with standard anti-inflammatory treatment, including persistent CMO Severe vitritis obstructing a clear view of the fundus Worsening intraocular inflammation in the absence of an identifiable underlying cause
	Combined with other PPV indications: Diagnostic vitrectomy in cases of suspected infections or masquerade syndromes Treatment of complications arising from uveitis: Persistent vitreous opacities Complex or complicated cataract Epiretinal membrane Rhegmatogenous retinal detachment Vitreous hemorrhage Tractional retinal detachment
Contraindications	Pre-existing hypotony, unless surgical intervention is needed to remove ciliary membranes or address ciliary atrophy with silicone oil injection Paediatric uveitis, generally avoided in children under 16 years because of the risk of anterior vitreous membrane formation and early onset of nuclear sclerosis; exceptions include cases combined with cataract surgery for complicated cataracts or isolated intermediate uveitis/pars planitis) Poor view of the posterior segment due to corneal opacities or cataract

Abbreviations: CMO: cystoid macular oedema; JIA, Juvenile Idiopathic Arthritis; PPV, pars plana vitrectomy.

**Table 3:** Perioperative evaluation and management for therapeutic pars plana vitrectomy in uveitis [9,14,21].

Preoperative evaluation and management	Clinical & laboratory assessment Determine anatomical classification of uveitis based on primary site of inflammation Grade anterior chamber cells and vitreous haze Perform ocular imaging to assess disease activity and extent Conduct targeted laboratory tests to identify the etiological cause and rule out infectious or systemic disease Guides initial medical therapy, decision for therapeutic PPV, and postoperative care
	Anatomical evaluation Confirm adequate media clarity for intraoperative visualization Measure IOP to rule out hypotony Perform B-scan ultrasonography for dense vitritis or suspected RD, fibrous bands, or choroidal masses Use ultrasound biomicroscopy for ciliary body assessment in hypotony Preoperative gonioscopy recommended even with normal IOP to assess risk of postoperative IOP elevation.
Intraoperative technique	Surgical entry Use small-gauge instruments (23/25/27 G); sharp trocars to avoid detachment of inflamed pars plana 27 G may prolong surgery in very viscous vitreous.
	Vitreous management High cut rate (≥5000–10,000 cpm) with low suction (≤300 mm Hg). Most cases have complete PVD; trim vitreous adhesions if PVD incomplete Post-vitrectomy fundus evaluation to detect chorioretinal lesions or intraoperative complications. Consider complete vitreous base dissection in paediatric cases to prevent ciliary body membranes
Additional interventions	Lens management via anterior or pars plana route. ERM removal Laser photocoagulation or cryotherapy Air, gas, or silicone oil tamponade if indicated Document previously unrecognized pathologies (ERM, fibrovascular proliferation, retinal breaks).
Surgical closure	Partial fluid–air exchange to prevent postoperative hypotony Low threshold for transconjunctival suturing of ports if leakage suspected
Intraocular/periocular corticosteroids	Administer triamcinolone acetonide, fluocinolone acetonide, or sustained-release dexamethasone implants to augment anti-inflammatory effect Ensure no history of steroid response before use
Postoperative management	Control surgery-induced inflammation with topical and/or systemic corticosteroids Continue long-term immunosuppressive therapy for non-infectious uveitis as indicated Adjust immunosuppressive doses; PPV may allow reduction Treat infectious uveitis with targeted antimicrobial therapy Monitor for retinal breaks, detachment, CMO, ERM, macular holes, CNVM, IOP changes, and cataract progression Frequency of follow-up depends on severity of inflammation and aetiology

Abbreviations: CMO, cystoid macular oedema; CNVM, choroidal neovascular membranes; ERM, epiretinal membrane; G, gauge; IOP, intraocular pressure; PPV, pars plana vitrectomy; PVD, posterior vitreous detachment; RD, retinal detachment.

**Table 4:** Summary of studies on PPV for ERM in uveitis.

Authors (Year)	Study Design/Number of Eyes	Surgical Approach	Follow-Up	Key Visual Outcomes	Anatomical Outcomes	Effect on Uveitis Activity/Comments
Yap et al. (2024) [118]	Retrospective cohort; 216 eyes with ERM (44 operated)	ERM peel	12 months (surgical subgroup)	VA improved from 20/60 to 20/40; 61% improved	Low ERM progression rate (7.9%)	Identified ERM risk factors; good long-term prognosis
El Faouri et al. (2023) [106]	Retrospective single-centre; 27 eyes	PPV without macular intervention	Up to 24 months	78% improved VA	88% resolution of macular oedema	Reduced inflammation and systemic therapy
Cristescu et al. (2022) [100]	Bicentre retrospective; 29 eyes	PPV + ERM peel ± ILM peel	Mean 32 months	BCVA improved 0.73 → 0.49 logMAR	CRT reduced (456 → 353 µm); CMO reduced	ILM peel not determinant; minimal uveitis reactivation
Coassin et al. (2021) [103]	Retrospective case series; 26 eyes	PPV + ERM + ILM peel (quiescent uveitis)	Mean 67 months	VA improved 20/80 → 20/40; ≥2 ETDRS lines in 54%	CFT reduced (428 → 328 µm)	Emphasized surgery during inactive inflammation
Rao et al. (2018) [115]	Multicentre retrospective; 17 eyes	23G PPV + ERM + ILM peel	Mean 23 months	Trend toward VA improvement (0.8 → 0.6 logMAR)	CFT reduced (517 → 371 µm); no ERM recurrence	All eyes inactive at final visit; 29% required additional immunotherapy
Miranda et al. (2016) [117]	Retrospective case series; 14 eyes (toxoplasmosis)	PPV for ERM	Mean 6 months	VA improved from 20/200 to 20/60	OCT anatomy improved	No recurrences; cataract and PCO most common complications
Tanawade et al. (2015) [104]	Retrospective case series; 16 eyes	PPV + ERM peel ± ILM peel	≥6 months	VA improved 31%, stable 31%, worsened 38%	Variable outcomes	Better outcomes with macular traction; cataract and macular pathology affected results
Kiryu et al. (2003) [116]	Retrospective case series; 11 eyes (sarcoidosis)	PPV for ERM	Up to ≥12 months	45% gained ≥ 2 Snellen lines at final FU; 81% ≥20/40	CMO resolved in 4/7 eyes	Cataract and ERM recurrence limited final VA

Abbreviations: BCVA, best-corrected visual acuity; CFT, central foveal thickness; CMO, cystoid macular oedema; CRT, central retinal thickness; ETDRS, Early Treatment Diabetic Retinopathy Study; ERM, epiretinal membrane; FU, follow-up; ILM, internal limiting membrane; OCT, optical coherence tomography; PCO, posterior capsule opacification; PPV, pars plana vitrectomy; VA, visual acuity.

**Table 5:** Summary of uveitic macular hole surgery outcomes.

Author (Year)	Aetiology/Population	Eyes	Mean Age	MH Type/Pathogenesis	Surgical Technique	MH Closure Rate	Visual Acuity Outcomes	Key Complications/Limitations
Macky et al. (2025) [132]	Active ocular Behçet’s disease	15 eyes (14 pts)	29.3 yrs	Full-thickness MH; severe ERM, inflammation, ischemia; some with RD	PPV + ERM removal + ILM peel + gas or silicone oil	93% (14/15)	Mean BCVA: 0.04 → 0.15 (3 mo), declined to 0.06 at 6 mo	Recurrent inflammation, macular ischaemia limited long-term VA
Callaway et al. (2018) [131]	Multicentre uveitis (incl. viral retinitis)	20 eyes (19 pts)	NR	Inflammatory full-thickness MH	PPV + ICG-assisted ILM peel	81% (at 3 mo)	Mean VA: 20/200 → 20/63 at 3 mo; ≥2-line gain in 75%	Persistent inflammation; some non-closure despite surgery
Sousa et al. (2021) [129]	Toxoplasmosis-related MH	11	33.2 ± 11 yrs	Full-thickness MH adjacent to inactive toxoplasmosis scars	PPV + ERM peel (if present) + ILM peel	100%	Mean VA: 20/252 → 20/54 (final); ≥3-line gain in 100%	Cataract in 1 eye; otherwise excellent safety
Hirano et al. (2015) [130]	Posterior uveitis (case report)	1	80 yrs	Large chronic full-thickness MH (minimum diameter 569 μm) with post-inflammatory chorioretinal atrophy	PPV + inverted ILM flap technique + gas, assisted by low–molecular-weight hyaluronic acid	100% (single case)	Anatomical closure by postoperative Day 3; VA outcome not emphasized	Single case; extensive chorioretinal atrophy; innovative technique for large/refractory MH
Woo et al. (2009) [128]	Uveitis (Behçet’s, CMV retinitis, idiopathic posterior/intermediate uveitis)	7	46 ± 13 yrs	Full-thickness MH; mainly tractional from ERM (5/7), CME-related in others	20G PPV + gas (C3F8); ILM peel in 6/7	100%	Mean VA: 20/554 → 20/294 (3 mo) → 20/444 (final); 86% improved at 3 mo	High postoperative IOP in all eyes; severe glaucoma in 3 eyes; limited final VA

Abbreviations: BCVA, best-corrected visual acuity; CME, cystoid macular oedema; CMV, cytomegalovirus; C3F8, perfluoropropane gas; ERM, epiretinal membrane; ICG, indocyanine green; ILM, internal limiting membrane; IOP, intraocular pressure; MH, macular hole; PPV, pars plana vitrectomy; RD, retinal detachment; VA, visual acuity.

## Data Availability

No new data were created or analysed in this study. Data sharing is not applicable.
